# Proximity-dependent biotin labeling in testicular germ cells identified TESMIN-associated proteins

**DOI:** 10.1038/s41598-022-26501-7

**Published:** 2022-12-23

**Authors:** Seiya Oura, Akinori Ninomiya, Fuminori Sugihara, Martin M. Matzuk, Masahito Ikawa

**Affiliations:** 1 Department of Experimental Genome Research, Research Institute for Microbial Diseases, Osaka University, Osaka, 565-0871 Japan; 2 Graduate School of Pharmaceutical Sciences, Osaka University, Osaka, 565-0871 Japan; 3 Core Instrumentation Facility, Research Institute for Microbial Diseases, Osaka University, Suita, Osaka 565-0871 Japan; 4 Department of Pathology & Immunology, Baylor College of Medicine, Houston, TX 77030 USA; 5 Center for Drug Discovery, Baylor College of Medicine, Houston, TX 77030 USA; 6 The Institute of Medical Science, The University of Tokyo, Minato-Ku, Tokyo 108-8639 Japan; 7 Center for Infectious Disease Education and Research (CiDER), Osaka University, Osaka, 565-0871 Japan

**Keywords:** Spermatogenesis, Protein-protein interaction networks

## Abstract

Characterization of protein–protein interactions (PPI) is a key to understanding the functions of proteins of interest. Recently developed proximity-dependent biotin identification (BioID) has been actively investigated as an alternative PPI mapping method because of its usefulness in uncovering transient PPI. Here, as an example of proximity labeling proteomics application in the testis, we generated two transgenic mouse lines expressing two biotin ligases (BioID2 or TurboID) fused with TESMIN, which translocates from the cytosol to the nucleus during meiotic progression and is required for reproduction. The BioID2 transgene, albeit not the TurboID transgene, rescued fertility defects of the *Tesmin* KO male mice, indicating that the TESMIN-BioID2 fusion can physiologically replace TESMIN. Furthermore, biotinylated protein pull-down and affinity-purification followed by mass spectrometry using the TESMIN-BioID2 transgenic mice captured components of the MYBL1–MuvB complex that regulate cell-cycle gene expression. Thus, our study shows that proximity labeling proteomics can be applied in male germ cells, although the choice of biotin ligase needs to be carefully tested.

## Introduction

Proteins generally work as multi-component complexes, and these protein–protein interactions (PPI) are essential for their proper functions. Therefore, the identification of interacting partners is a fundamental approach to elucidating the role of proteins of interest. Over the past decades, affinity purification followed by mass spectrometry (AP-MS) has been the most often used method to characterize PPI^1^. Furthermore, the CRISPR/Cas9 system^2,3^ has reduced the time and cost of preparing negative controls (i.e., knockout animals) and affinity-tag knock-in animals, facilitating PPI characterization in animal models. AP-MS thus remains a versatile and fundamental approach. Still, in recent years, proximity-dependent biotin identification (BioID)^4–6^ methods have begun to be actively investigated because of their usefulness in identifying insoluble proteins and transient PPI^7,8^.

The proximity labeling proteomics methods rely on fusing a promiscuous biotin ligase with a protein of interest, which acts as bait. Upon the addition of excess biotin, the fused biotin ligase biotinylate neighboring prey proteins. Those biotinylated proteins can be purified using streptavidin coupled to a solid support after cell lysis. As streptavidin tetramer and biotin-streptavidin binding are highly stable^9–11^, harsh conditions such as 0.4% Sodium Dodecyl Sulfate (SDS) can be used for cell lysis in proximity labeling experiments^5,6^. Furthermore, covalent biotinylation before cell lysis in proximity labeling approaches enables us to capture weak and transient PPI, which are difficult to detect in AP-MS^12^. Due to these benefits, proximity labeling proteomics has been rapidly adopted for animal models such as mice^8,13–17^. So far, two improved biotin ligases, BioID2^5^ with slower kinetics and TurboID^4^ with faster kinetics, have been actively investigated for their adaptation. However, further consideration is still needed to examine whether the fused biotin ligase affects the protein of interest.

TESMIN, a testis expressed metallothionein-like protein, which is highly expressed in spermatocytes (Fig. S1)^18^, has been reported^19,20^ to localize to the cytoplasm of pachytene spermatocytes until stage IX of the seminiferous epithelial cycle^21,22^ and then to translocate into the nuclei of germ cells in stage X, corresponding to the appearance of diplotene spermatocytes. Although we previously showed male infertility of *Tesmin* knockout (KO) mice due to meiosis defects^23^, the mechanism and physiological importance of the TESMIN translocation remain unclear. Therefore, in this study, we generated two transgenic mouse lines expressing TESMIN fused with BioID2 or TurboID to fully decipher the TESMIN interactome.

## Results

### A BioID2 transgene rescued *Tesmin* KO male fertility

**Figure 1:**
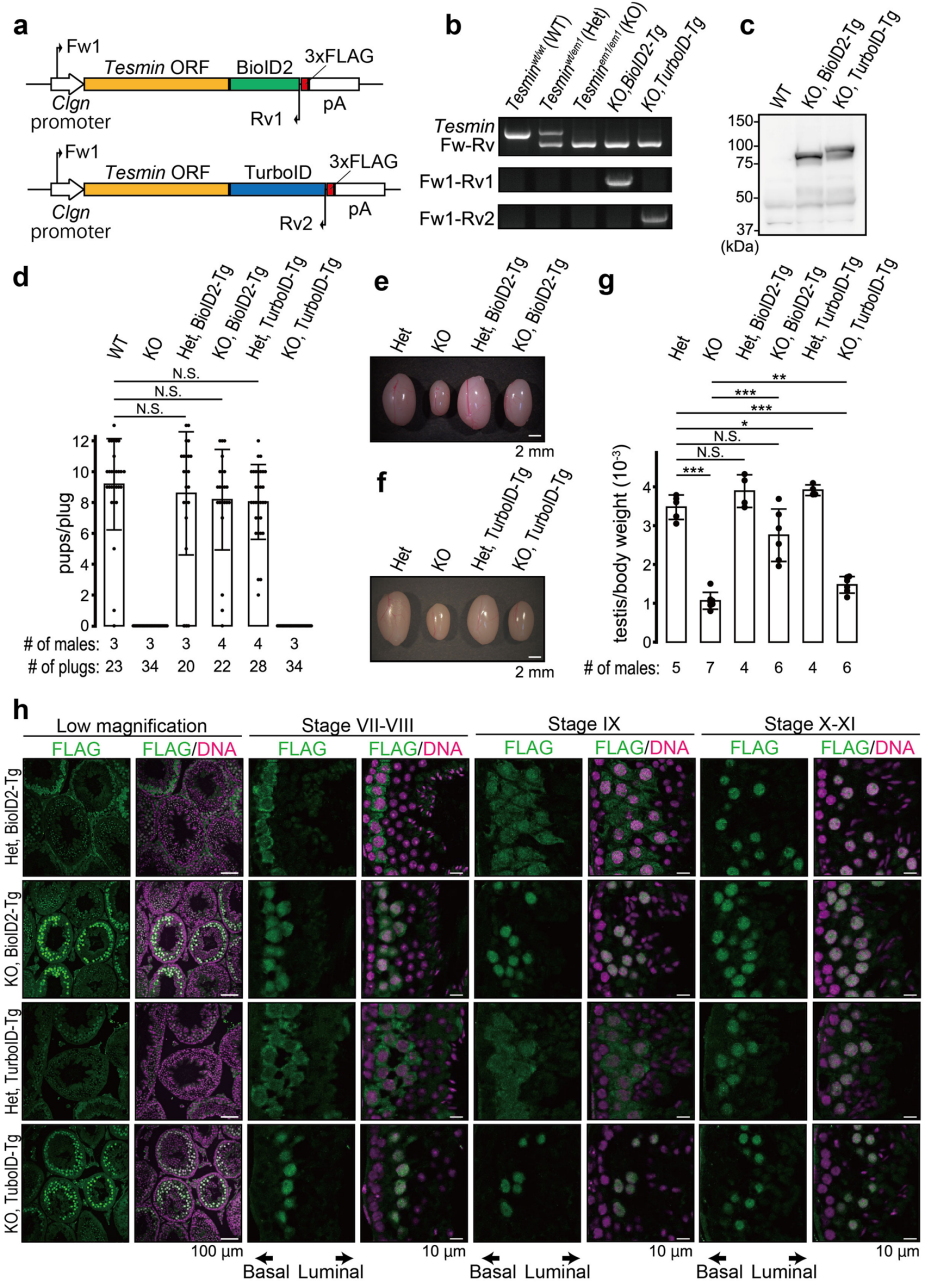
Production of BioID2 and TurboID transgenic mice and fertility analysis. (**a**) Schematic of Tg constructs. White arrows indicate Clgn promoter. Yellow boxes indicate Tesmin ORF (NM_001039657.2). Green and blue boxes indicate BioID2 and TurboID sequences, respectively. Red boxes indicate a 3xFLAG sequence. Black arrows indicate primers for genotyping. (**b**) An example of PCR genotyping with two primer sets shown in (**a**). (**c**) Immunoblot analysis with antibodies against FLAG. (**d**) Schematic of KCTD19 protein structure and antigen position. (**d**) The result of mating tests. Pups/plug: 8.9 ± 1.5 [WT]; 0 [KO]; 8.6 ± 4.0 [Het, BioID2-Tg]; 8.2 ± 3.3 [KO, BioID2-Tg]; 8.0 ± 2.4 [Het, TurboID-Tg]; 0 [KO, TurboID-Tg]. Error bars indicate standard deviations. p-value: 5.5 × 10^–1^ [WT and Het, BioID2-Tg]; 2.6 × 10^–1^ [WT and KO, BioID2-Tg]; 1.1 × 10^–1^ [WT and Het, TurboID-Tg]. (**e**–**g**) Testis morphology and (**e**, **f**) testis/bodyweight of adult mice (**g**). Testis/body weight: 3.4 ± 0.3 × 10^–3^ [Het]; 0.87 ± 0.2 × 10^–3^ [KO]; 3.9 ± 0.4 × 10^–3^ [Het, BioID2-Tg]; 2.8 ± 0.7 × 10^–3^ [KO, BioID2-Tg]; 3.9 ± 0.1 × 10^–3^ [Het, TurboID-Tg]; 1.5 ± 0.2 × 10^–3^ [KO, TurboID-Tg]. Error bars indicate standard deviations. p-value: 2.1 × 10^–8^ [Het and KO]; 1.3 × 10^–1^ [Het and Het, BioID2-Tg]; 5.6 × 10^–2^ [Het and KO, BioID2-Tg]; 3.7 × 10^–2^ [Het and Het, TurboID-Tg]; 5.2 × 10^–7^ [Het and KO, TurboID-Tg]; 6.0 × 10^–5^ [KO and KO, BioID2]; 5.6 × 10^–3^ [KO and KO, TurboID]. (**h**) Immunostaining of testis sections using anti-FLAG antibodies. The seminiferous epithelium cycle was determined by cell position, nuclear morphology, and morphology of the acrosome stained with AlexaFlour 568-conjugated lectin PNA.

We previously showed that a transgene expressing only the long isoform of TESMIN (TESMIN-L) rescues the meiotic defects and male fertility in *Tesmin* KO mice, whereas the small isoform of TESMIN (TESMIN-S) is not required for spermatogenesis^23^. Therefore, we used the TESMIN-L encoding sequence (ENSMUST00000025840.16) for transgenic mouse production and referred to it as TESMIN throughout this study. We injected a DNA construct having 3xFLAG-tagged *Tesmin*-BioID2 or *Tesmin*-TurboID under the control of the testis-specific *Clgn* promoter^24^ (Fig. 1a) and established transgenic (Tg) mouse lines (Fig. 1b). Tg expression was confirmed by immunoblot analysis using an anti-FLAG antibody (Fig. 1c). Subsequently, we caged the transgenic mice with WT females and observed a normal count of pups from Het Tg mice (Fig. 1d). Furthermore, gross testis morphology (Fig. 1e,f) and testis weight (Fig. 1g) were comparable between Het and Het-Tg mice, showing that TESMIN-BioID2 and TESMIN-TurboID expression themselves do not harm germ cells. The *Tesmin*-BioID2 transgene also rescued the fertility of KO male mice (Fig. 1d), although the testes were slightly smaller than Het counterpart (Fig. 1e,g, testis/body weight: 3.5 ± 0.3 × 10^–3^ [Het]; 2.8 ± 0.7 × 10^–3^ [KO, BioID2-Tg]). However, KO male mice carrying the *Tesmin*-TurboID transgene were infertile (Fig. 1d), despite a slight recovery of testicular weight (Fig. 1e,g, testis/body weight: 9.9 ± 2.8 × 10^–4^ [KO]; 1.5 ± 0.2 × 10^–3^ [KO, TurboID-Tg]).

### Histological and cytological analysis of transgenic mice

For a more detailed analysis of spermatogenesis, we performed hematoxylin and periodic acid-Schiff (HePAS) staining of testicular sections (Fig. S2a). Then, we compared testicular cells based on the cycle of the seminiferous epithelium^21,22^. As we previously reported^23^, spermatogenesis in *Tesmin* KO testis was predominantly arrested at an early stage of meiosis. Almost no spermatocytes existed in tubules after seminiferous stage IV–V (Fig. S2a; KO). On the other hand, spermatocytes and spermatids overall were present throughout the seminiferous epithelial cycles of KO male mice with *Tesmin*-BioID2 or *Tesmin*-TurboID transgenes (Fig. S2a; KO, BioID2-Tg; KO, TurboID-Tg), although the number of germ cells beyond the early pachytene stage was lower in *Tesmin*-TurboID positive KO males (Figs. S2a and S1b; KO, TurboID-Tg). Consistent with this result, both transgenes reduced the number of TUNEL (TdT-mediated dUTP nick-end labeling) positive germ cells (Fig. S3a,b). However, synapsis and XY body formation defects remained in *Tesmin*-TurboID positive KO males (Fig. S3c,d). These results demonstrated that both *Tesmin*-BioID2 or -TurboID transgenes rescued spermatogenesis, but the recovery by *Tesmin*-TurboID was partial. As a note, we did not observe any defects in the spermatogenesis of Het Tg males (Figs. S2 and S3).

We subsequently examined the localization patterns of TESMIN-BioID2 and TESMIN-TurboID by immunostaining testicular sections using an anti-FLAG antibody (Fig. 1h). In Het Tg mice, TESMIN-BioID2 and TESMIN-TurboID were mainly localized to the cytoplasm of pachytene spermatocytes in tubules before stage VIII, detected in both the cytoplasm and nuclei of spermatocytes at seminiferous stage IX–X, and were observed only in the nuclei of spermatocyte after stage X (Fig. 1h; Het, BioID2-Tg; Het, TurboID-Tg). These results showed the translocation of TESMIN from the spermatocyte cytoplasm to the nuclei at seminiferous stage IX, as previously reported^19^. However, TESMIN-BioID2 in the KO background existed in both the cytoplasm and nuclei of spermatocytes before seminiferous stage VIII and localized to the nuclei after stage IX (Fig. 1h; KO, BioID2-Tg). Furthermore, TESMIN-TuroboID in the KO background was always localized to the spermatocyte nuclei (Fig. 1h; KO, TurboID2-Tg), suggesting that the fusion of TurboID affected TESMIN localization patterns.

### Identification of TESMIN-associated proteins

**Figure 2:**
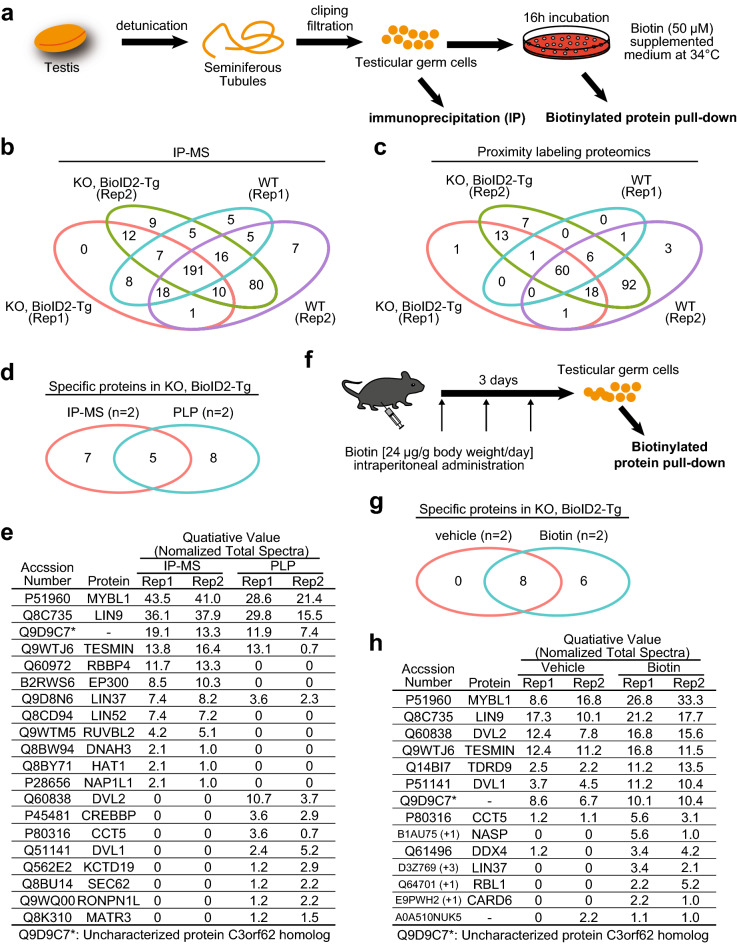
Interactome analysis of TESMIN. (**a**) Schematic overview of sample preparation for mass spectrometry following immunoprecipitation and biotinylated protein pull-down. (**b**, **c**) Venn diagram of IP-Mass (**b**) and proximity labeling proteomics (**c**) analysis. Two mice were used for each experimental group. (**d**) Venn diagram of proteins that were reproducibly captured in IP-MS (**b**) and proximity labeling proteomics (**c**) using KO, BioID2-Tg mice. Proteins detected at least once in the wild type are excluded (described as specific). (**e**) The list of specific proteins in d and quantitative values (normalized total spectra). The whole data is available in Dataset S1. (**f**) Schematic overview of intraperitoneal administration of biotin. (**g**) Venn diagram of proteins that were reproducibly captured in proximity labeling proteomics using vehicle- and biotin-injected KO, BioID2-Tg mice. Proteins detected at least once in the wild type are excluded (described as specific). (**h**) The list of specific proteins in g and quantitative values (normalized total spectra). The whole data is available in Dataset S1.

To characterize the TESMIN interactome, we performed immunoprecipitation using an anti-FLAG antibody and biotinylated protein pull-down using streptavidin (Fig. 2a), followed by mass spectrometry (IP-MS and proximity labeling proteomics, respectively; Dataset S1). We detected some proteins specifically in BioID2-Tg positive KO, not in WT (Fig. 2b,c). Notably, some proteins were detected by only one of the methods (Fig. 2d), as previously reported^12^. In detail, MYBL1 and 4 out of 5 MuvB core complex^25^, RBBP4, LIN9, LIN37, and LIN52 were reproducibly detected in IP-MS (Fig. 2e). Alternatively, proximity labeling proteomics captured only MYBL1 and LIN9 reproducibly, which is consistent with the proximity-dependent nature. More clearly, disheveled segment polarity protein 1 (DVL1) and DVL2, which are implicated in nuclear-cytoplasmic translocation^26–28^, were detected only in the proximity labeling approach (Fig. 2e).

Because we were concerned about artifacts of incubation in a biotin-supplemented medium, we performed proximity labeling using fresh TGCs collected from BioID2-Tg positive KO mice fed with a standard laboratory rodent diet (containing 0.514 ppm biotin, Fig. S4a–c). As a result, we successfully captured MYBL1, LIN9, and DVL2 in fresh TGCs (Fig. S4c), although the total spectrum counts tended to be lower than incubated TGCs. Then we also consider intraperitoneal administration of biotin. We injected biotin at 24 μg/g body weight^15^ for 3 days and harvested TGCs (Fig. 2f). As a result, we captured more protein with higher quantitative value in biotin-injected BioID2-Tg positive KO mice than vehicle-injected ones (Fig. 2g,h).

When we performed proximity labeling proteomics using BioID2-Tg positive Het TGCs, incubated in the biotin supplemented medium, we detected similar proteins with BioID2-Tg positive KO TGCs (Fig. S4d–g), ruling out the possibility that abnormal localization of TESMIN-BioID2 disturbed its interatomic pattern. Then, we also considered *Tesmin*-TurboID Tg mice (Fig. S5). Harvested TGCs were incubated in a 500 μM biotin supplemented medium for 1 h and subjected to biotinylated protein pull-down. As a result, we successfully detected MYBL1 and LIN9 in TurboID-Tg positive TGCs as well as in BioID2-Tg positive TGCs (Fig. S5c and S5e), although we could not capture DVL1/DVL2 (Fig. S5c,e).

**Figure 3:**
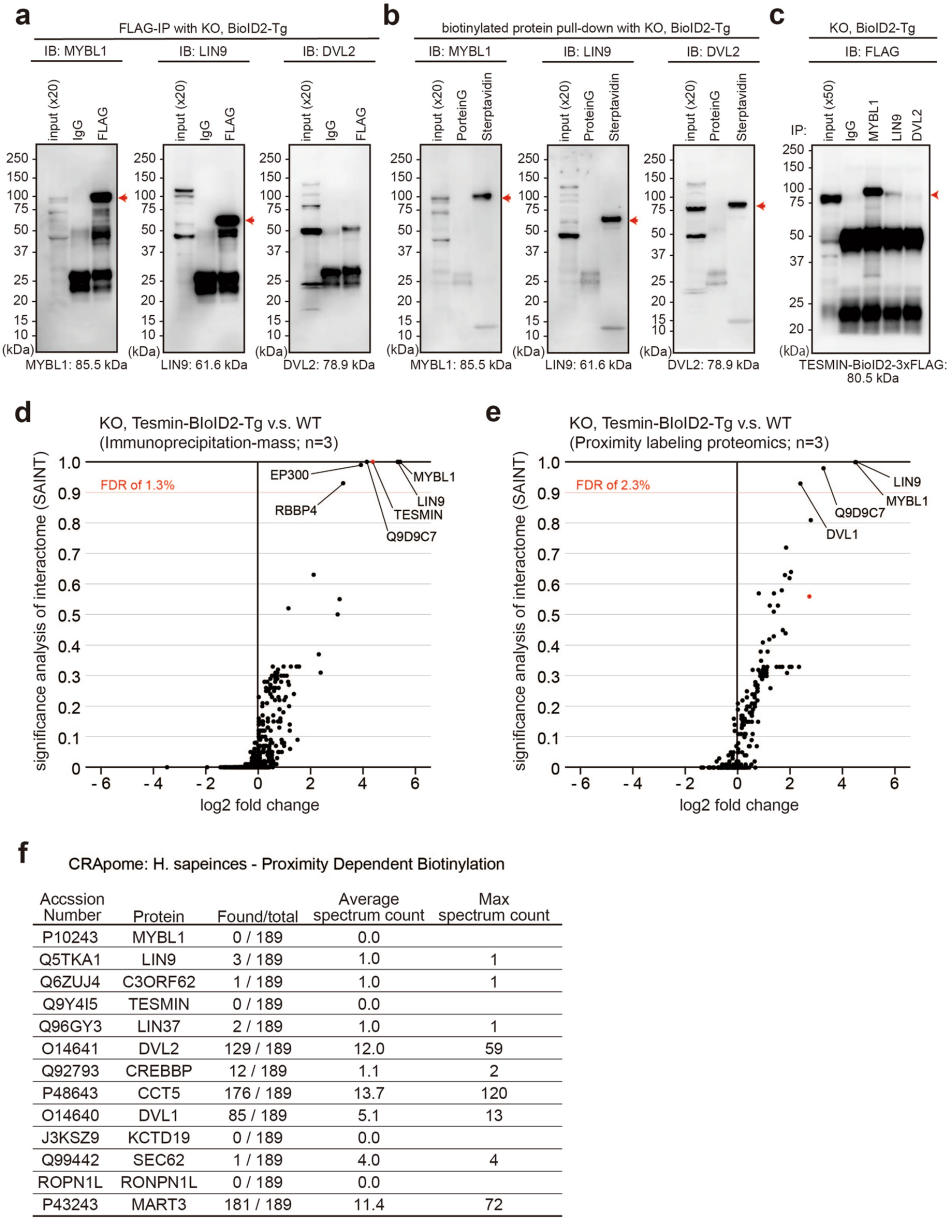
Immunoprecipitation and immunostaining of representative proteins. (**a**, **b**) Immunoblot analysis for MYBL1, LIN9, and DVL2 using eluted samples of IP (**a**) and proximity labeling proteomics (**b**). Red arrows indicate the expected protein size. (**c**) Immunoblot analysis for TESMIN-BioID2-3xFLAG using eluted samples of immunoprecipitation using anti-MYBL1, -LIN9, and -DVL2 antibodies. (**d**, **e**) Significance analysis of interactome (SAINT) and log2 fold change of average spectrum count are shown as scatterplots for immunoprecipitation-mass (**d**) and proximity labeling proteomics (**e**). The led dots indicate TESMIN. (**f**) The human orthologs of mouse proteins that were captured in proximity labeling proteomics using Tesmin-BioID2 transgenic mice (Fig. 2e). Human data were referred to because no mouse data are deposited. The found/total column shows the number of proximity labeling experiments that captured each protein in the contaminant repository for affinity purification (CRAPome) database.

We confirmed MYBL1 and LIN9 association with TESMIN by immunoblot analysis followed by IP and SA-pull down. DVL2 was only captured in biotinylated protein pull-down (Fig. 3a–c), consistent with the results of Mass spectrometry. Furthermore, significance analysis of interactome (SAINT)^29^ called MYBL1, LIN9, and Q9D9C7 at the probability threshold of 0.9, which was approximately equivalent to an estimated false discovery rate (FDR) of less than 3%, from both IP-MS and proximity labeling proteomics using BioID2-Tg positive KO mice (Fig. 3d,e). DVL proteins were selected only in proximity labeling proteomics (Fig. 3e and Dataset S2). To further study the TESMIN interacting proteins, we examined the localization of MYBL1, LIN9, and DVL2 in WT testis by immunostaining. MYBL1 and LIN9 were detected in the spermatocyte nuclei (Fig. S6), irrespective of the TESMIN localization change at seminiferous stage IX. In contrast, DVL2 existed in both the cytoplasm and nuclei of spermatocytes before stage VIII and localized to the nuclei after stage X (Fig. S6), reminiscent of TESMIN translocation. However, DVL2 was found in 129 out of 189 proximity labeling proteomics experiments deposited in the contaminant repository for affinity purification database (CRAPome; https://reprint-apms.org/; accessed on Jul 23, 2022; Fig. 3f)^30^, indicating DVL2 might be a contaminant of proximity labeling proteomics using BioID2 enzyme.

## Discussion

To capture the TESMIN interactome, we performed both IP-MS and proximity labeling proteomics. Briefly, IP-MS detected almost all components of the MYBL1–MubV complex reproducibly. In contrast, the proximity labeling method captured only some members of the complex, MYBL1, LIN9, and LIN37, which are expected to locate in the vicinity of TESMIN. Zhang et al. recently reported IP-MS analysis against TESMIN and association with MYBL1–MuvB complex^20^, corroborating our results. These results confirmed that proximity labeling proteomics is applicable in testicular germ cells.

This study examined two promising biotin ligases: BioID2^6^ and TurboID^4^. However, only the *Tesmin*-BioID2 transgene rescued the fertility of *Tesmin* KO male mice. Considering that the physiological concentration of biotin was enough for their biotin ligase activity, TurboID with faster kinetics might disturb the function and localization of TESMIN because of self-biotinylation. This hypothesis is corroborated by the observation of *Tesmin*-BioID2 transgenic KO male mice: BioID2 with slower kinetics partially affected the localization of TESMIN and caused milder spermatogenic defects than TurboID. Although the shorter biotin-labeling time window of TurboID is beneficial for in vivo and ex vivo applications, both BioID2 and TurboID should be tested. As a note, TurboID-Tg positive heterozygous KO mice were fully fertile, ruling out the possibility TurboID expression itself was detrimental to germ cells.

In a previous report, LIN9 was suggested to be implicated in TESMIN translocalization^20^. However, TESMIN remains in the spermatocyte cytoplasm in the tubules before stage VIII, regardless of the nuclear localization of LIN9 (Fig. S6). In this study, we identified DVL1/DVL2 as TESMIN-associated proteins. The DVL proteins are involved in protein shuttle systems such as YAP nuclear exportation^28^, suggesting that LIN9-mediated nuclear importation might be canceled by DVL1/DVL2 before stage VIII, although we could not rule out the possibility that DVL1/DVL2 might be a contaminant. To examine whether DVL1/DVL2 is related to TESMIN translocation, testis-specific conditional KO of *Dvl1*/*Dvl2* needs to be considered because most *Dvl2* KO male mice failed to survive until adulthood ^31^. Further, free BioID2/TurboID expressing animals should be used as a negative control in the future proximity labeling proteomics to rule out the possibility of direct interaction of captured proteins and BioID2/TurboID.

Thus, our study showed that proximity labeling proteomics can be applied to male germ cells in mice, although the choice of biotin ligase needs to be carefully tested. Accumulation of in vivo proteomics data will help to distinguish contaminants from the captured spectrum, further expanding the applicability of proximity labeling proteomics.

## Material and methods

### Animals

B6D2F1 (C57BL/6 × DBA2; Japan SLC, Shizuoka, Japan), C57BL6/N (SLC), ICR (SLC), and *Tesmin* KO mice^23^ (B6D2-Mtl5 $\left\langle {{\text{em1Osb}}} \right\rangle$) were used in this study. *Tesmin*-deficient frozen spermatozoa are available from both the Riken BioResource Center (Riken BRC, Tsukuba, Japan) and the Center for Animal Resources and Development (CARD, Kumamoto, Japan) at Kumamoto University (Table S1). Animals were housed in a temperature-controlled environment with 12 h light cycles and free access to food (CE-2 sterilized with 20 kGy, CLEA Japan, Tokyo, Japan) and water. Mice were sacrificed by cervical dislocation by trained researchers for tissue sampling and embryo collection.

### Generation of *Tesmin*-BioID2-3xFLAG and *Tesmin*-TurboID-3xFLAG transgenic mice

The mouse *Tesmin* cDNA (ENSMUST00000025840.16) was tagged BioID2-3xFLAG or TurboID-3xFLAG with a rabbit polyA signal inserted under the control of CAG promoter. After confirmation of enzymatic activity in HEK293T culture cells, the promoter sequence was replaced by the mouse *Clgn* promoter for transgenic mouse production. These original and *Tesmin* cDNA inserted plasmids will be available from both the Riken BRC DNA bank (RBD) and Addgene (Table S1). After linearization, the DNA constructs (2.16 ng/µL; 0.54 ng/µL/kbp) were injected into the pronucleus of fertilized eggs. The following day, the injected eggs were transplanted into the oviduct ampulla of pseudopregnant mice (ICR; 10 embryos per ampulla). After 19 days, pups were delivered through Caesarean section and placed with foster mothers (ICR). Offspring carrying *Tesmin*-BioID2-3xFLAG and *Tesmin*-TurboID-3xFLAG were used in this study. The genotyping primers (GeneDesign, Osaka, Japan) are available in Table S1. Gene-manipulated mouse lines used in this study are deposited at both Riken BRC and CARD (Table S1). All lines will be available through these centers.

### Immunoblot analysis

Proteins from testis were extracted using NP40 lysis buffer [50 mM Tris–HCl (pH 7.5), 150 mM NaCl, 0.5% NP-40, 10% Glycerol]. Proteins were separated by SDS-PAGE under reducing conditions and transferred to polyvinylidene fluoride (PVDF) membrane using the Trans-Blot Turbo system (BioRad, Munich, Germany). After blocking with 10% skim milk (232100, Becton Dickinson, Cockeysville, MD, USA), the membrane was incubated with primary antibody overnight at 4 °C, and then incubated with HRP-conjugated secondary antibody for 1 h at room temperature. Chemiluminescence was detected by ECL Prime Western Blotting Detection Reagents (RPN2232, GE Healthcare, Chicago, IL, USA) using the Image Quant LAS 4000 mini (GE Healthcare). The antibodies used in this study are listed in Table S1.

### Fertility analysis

To examine fertility, sexually mature male mice were caged with wild-type females (B6DF1) for at least three months. The vaginal plugs and pup numbers were recorded at approximately 10 AM to determine the number of copulations and litter size. Numerical data is available in Table S2.

### Morphological and histological analysis of testis

To observe testis gross morphology and measure testicular weight, mice over 12 weeks of age were euthanized after measuring their body weight. Numerical data is available in Table S2. The whole testis was observed using BX50 and SZX7 (Olympus, Tokyo, Japan) microscopes. For histological analysis, testes were fixed with Bouin's fixative (16045-1, Polysciences, Warrington, PA, USA) at 4 °C O/N, dehydrated in increasing ethanol concentrations and 100% xylene, embedded in paraffin, and sectioned (5 µm). The paraffin sections were hydrated with Xylene and decreasing ethanol concentrations and treated with 1% periodic acid (26605-32, Nacalai Tesque, Kyoto, Japan) for 10 min, treated with Schiff's reagent (193-08445, Wako) for 20 min, counterstained with Mayer's hematoxylin solution (131-09665, Wako) for 3 min, dehydrated in increasing ethanol concentrations, and finally mounted with Permount (SP15-100-1, Ferma, Tokyo, Japan). The sections were observed using a BX53 (Olympus) microscope. Seminiferous tubule stages were identified based on the morphological characteristics of the germ cell nuclei^32^.

### Apoptosis detection in testicular section

TdT-mediated dUTP nick end labeling (TUNEL) staining was carried out with In Situ Apoptosis Detection Kit (MK500, Takara Bio Inc., Shiga, Japan), according to the manufacturer’s instruction. Briefly, testes were fixed with Bouin's fixative, embedded in paraffin, and sectioned (5 µm). After paraffin removal, the slides were boiled in citrate buffer (pH 6.0; 1:100; ab93678, abcam, Cambridge, UK) for 10 min and incubated in 3% H_2_O_2_ at room temperature for 5 min for endogenous peroxidase inactivation, followed by a labeling reaction with TdT enzyme and FITC-conjugated dUTP at 37 °C for 1 h.

For chromogenic detection of apoptosis, the sections were incubated with HRP-conjugated anti-FITC antibody at 37 °C for 30 min. The section was then incubated in ImmPACT DAB (SK-4105, Vector Laboratories, Burlingame, CA, USA) working solution, counterstained with Mayer's hematoxylin solution for 3 min, dehydrated in increasing ethanol concentrations, and finally mounted with Permount. The sections were observed using a BX53 (Olympus) microscope. Seminiferous tubule stages were identified based on the morphological characteristics of the germ cell nuclei^32^. Numerical data is available in Table S2.

### Immunostaining of testes

Testes were fixed in 4% paraformaldehyde (PFA) overnight at 4 °C, followed by dehydration in increasing ethanol concentrations and 100% of xylene, embedded in paraffin, and sectioned (5 µm). After paraffin removal, the slides were boiled in pH 6.0 citrate buffer for 10 min, blocked and permeabilized with 10% goat serum and 0.1% Triton X-100 for 20 min in PBS, and incubated with primary antibody overnight at 4 °C or 1 h at room temperature in blocking solution. After incubation with Alexa Flour 488/546-conjugated secondary antibody (1:200) at room temperature for 1 h, samples are counterstained with Hoechst 33342 (1:2000; H3570, Thermo Fisher Scientific) and mounted with Immu-Mount (9990402, Thermo Fisher Scientific). The antibodies used in this study are listed in Table S1.

Seminiferous tubule stages were identified based on the morphological characteristics of the germ cell nuclei and acrosome staining with Alexa Flour 488/568-conjugated lectin PNA (L21409/L32458, Thermo Fisher Scientific). The sections were observed using a BX53 (Olympus) microscope and a Nikon Eclipse Ti microscope connected to a Nikon C2 confocal module (Nikon, Tokyo, Japan). Fluorescent images were false-colored and cropped using ImageJ Fiji software.

### Immunostaining of surface chromosome spread

Spread nuclei from spermatocytes were prepared as previously described^33^. Seminiferous tubules were unraveled using forceps in ice-cold DMEM (11995065, Thermo Fisher Scientific) and incubated in 1 mg/mL collagenase (C5138, Sigma-Aldrich) in DMEM (20 mL) at 37 °C for 15 min. After 3 washes with DMEM, the tubules were transferred to 20 mL trypsin/DNaseI medium [0.025 w/v% trypsin, 0.01 w/v% EDTA, 10U DNase in DMEM] and incubated at 37 °C for 10 min. After adding 5 mL of heat-inactivated FCS and pipetting, the solution was filtered through 59 µm mesh (N-N0270T, NBC Meshtec inc., Tokyo, Japan) to remove tubule debris. The collected testicular cells were resuspended in hypotonic solution [100 mM sucrose] and 10 µL of the suspension was dropped onto a slide glass with 100 µL of fixative solution [100 µL of 1% PFA, 0.1% (v/v) Triton X-100]. The slides were then air-dried and washed with PBS containing 0.4% Photo-Flo 200 (1464510, Kodak Alaris, NY, USA) or frozen for longer storage at – 80 °C.

The spread samples were blocked with 10% goat serum in PBS and then incubated with primary antibodies overnight at 4 °C in blocking solution. After incubation with AlexaFlour 488/546-conjugated secondary antibody (1:200) at room temperature for 1 h, samples are counterstained with Hoechst 33342 and mounted with Immu-Mount. The samples were observed using a BX53 (Olympus) microscope. The antibodies used in this study are listed in Table S1. Numerical data is available in Table S2.

### Immunoprecipitation

Proteins were extracted using NP40 lysis buffer [50 mM Tris–HCl (pH 7.5), 150 mM NaCl, 0.5% NP-40, 10% Glycerol]. Protein lysates were mixed with 20 μL Protein G-conjugated magnetic beads (DB10009, Thermo Fisher Scientific) with 2.0 μg antibody. The immune complexes were incubated for 1 h at 4 °C and washed 3 times with NP40 lysis buffer. Co-immunoprecipitated products were then eluted by resuspension in 2 × SDS sample buffer [125 mM Tris–HCl (pH6.8), 10% 2-mercaptoethanol, 4% sodium dodecyl sulfate (SDS), 10% sucrose, 0.01% bromophenol blue] and 10 min incubation at 70 °C. The antibodies used in this study are listed in Table S1.

### Incubation in biotin supplemented medium

For preparing testicular germ cells (TGCs), seminiferous tubules were unraveled using forceps in ice-cold PBS and transferred to a 1.5-mL tube with 1 mL of accutase (12679-54, Nacalai Tesque), followed by clipping the tubules, a 5 min incubation at room temperature. After filtration with a 59 µm mesh and centrifugation, TGCs were suspended in an organ culture medium [2.02 g α-MEM (12000022, Thermo Fisher Scientific), 20 mL KSR (10828010, Thermo Fisher Scientific), 2 mL Antibiotic–Antimycotic (15240096, Thermo Fisher Scientific), 0.364 g Sodium bicarbonate, up to 200 ml with DDW]^34^ supplemented with 50 µM and 500 µM Biotin (B4501, Sigma-Aldrich) followed by 16 h and 1 h incubation at 34 °C for BioID2 and TurboID, respectively. Biotin was dissolved in DMSO at 100 mg/mL, stored at − 30 °C, and diluted at the time of use. After 3 washes with PBS, TGCs were incubated in NP40 lysis buffer [50 mM Tris–HCl (pH7.5), 150 mM NaCl, 0.5% NP-40, 10% Glycerol] for 20 min at 4 °C with gentle agitation for protein extraction.

### Intraperitoneal administration of biotin

The freeze-thawed 100 mg/mL biotin dissolved in DMSO was diluted 100 times in PBS immediately before use. The 1 mg/mL biotin solution was intraperitoneally injected into animals once daily at 24 μg/g body weight^15^ for 3 days.

### Biotinylated protein pull-down

Biotinylated protein pulldown was performed as previously described^35^ with slight modification. Briefly, protein lysates were mixed with 50 µL SA-conjugated magnetic beads (DB65001, Thermo Fisher Scientific), followed by 1 h incubation at 4 °C with gentle agitation. Beads were washed once with 1 mL wash solution 1 [2% SDS], once with 1 mL wash solution 2 [50 mM HEPES–NaOH (pH7.5), 500 mM NaCl, 1 mM EDTA, 0.1% sodium deoxycholate, 1% Triton X-100], once with 1 mL wash solution 3 [10 mM Tris–HCl (pH7.4), 250 mM LiCl, 1 mM EDTA, 0.1% sodium deoxycholate, 1% NP40]. Biotinylated proteins were then eluted by resuspension in 2 × SDS sample buffer [125 mM Tris–HCl (pH6.8), 10% 2-mercaptoethanol, 4% sodium dodecyl sulfate (SDS), 10% sucrose, 0.01% bromophenol blue] and 5 min incubation at 95 °C.

### Mass spectrometry and data analysis

Protein samples were first subjected to chloroform/methanol precipitation to remove SDS. Then, the dried pellets were dissolved in 20 μL of 0.1% RapiGest (Waters, Milford, MA, USA) and reduced with 10 mM dithiothreitol (DTT), followed by alkylation with 55 mM iodoacetamide, and digested by treatment with trypsin and purified with a C18 tip (GL-Science, Tokyo, Japan). The resultant peptides were subjected to nanocapillary reversed-phase LC–MS/MS analysis using a C18 column (25 cm × 75 µm, 1.6 µm; IonOpticks, Victoria, Australia) on a nanoLC system (Bruker Daltoniks, Bremen, Germany) connected to a tims TOF Pro mass spectrometer (Bruker Daltoniks) and a modified nano-electrospray ion source (CaptiveSpray; Bruker Daltoniks). The mobile phase consisted of water containing 0.1% formic acid (solvent A) and acetonitrile containing 0.1% formic acid (solvent B). Linear gradient elution was carried out from 2 to 35% solvent B for 18 min at a flow rate of 400 nL/min. The ion spray voltage was set at 1.6 kV in the positive ion mode. Ions were collected in the trapped ion mobility spectrometry (TIMS) device over 100 ms and MS and MS/MS data were acquired over an *m/z* range of 100–1700. During the collection of MS/MS data, the TIMS cycle was adjusted to 1.1 s and included 1 MS plus 10 parallel accumulation serial fragmentation (PASEF)-MS/MS scans, each containing on average 12 MS/MS spectra (> 100 Hz), and nitrogen gas was used as the collision gas.

The resulting data were processed using DataAnalysis version 5.1 (Bruker Daltoniks), and proteins were searched using MASCOT (Matrix Science, London, UK) against the SwissProt database. The MASCOT search results were filtered by Scaffold version 5 (Proteome Software, Portland, OR, USA; Protein Threshold: 99%; Min # Peptides: 2; Peptide Threshold: 95%). The quantitative values (Normalized Total Spectra) are available in Dataset S1. Venn diagrams were created with ggVennDiagram ver. 1.1.0. SAINT and Log2 fold change was calculated by using an analyze pipeline of the Resource for Evaluation of Protein Interaction Networks (REPRINT; https://reprint-apms.org/). Analysis parameters for SAINT are as follows: Choice of Controls: user controls, Combining Replicates: average, #Number of virtual controls: 10, n-burn: 2000, n-iter: 4000; minFold: 1, lowMode: 0, Normalize: 1. Analysis parameters for the emperical fold change score are as follows: Choice of Controls: user controls, Combining Replicates: average, #Number of virtual controls: 10. The overall results are available in Dataset S2. The Bayesian FDR was approximated as previously described^29^.

### Ethics statement and reproducibility

All animal experiments were approved by the Animal Care and Use Committee of the Research Institute for Microbial Diseases, Osaka University (#Biken-AP-R03-01-0), and all experiments were performed in accordance with these guidelines. This study is reported in accordance with the ARRIVE guidelines. Sample size and statistical methods were described in each legend or/and in the figure panel. P-value was calculated using R ver. 4.0.2.

## Supplementary Information

Dataset S1.

Dataset S2.

Supplementary Information 1.

## Data Availability

The authors declare that the data that support the findings of this study are available from the corresponding author upon request. Numerical data for graphs are listed in Table S2 and Dataset S1 and S2. The uncropped gel and blot images are available in Figs. S7 and S8. The deposition ID numbers for mouse lines and plasmids used in this study are listed in Table S1. The The mass spectrometry proteomics data have been deposited to the ProteomeXchange Consortium via the PRIDE^36^ partner repository with the dataset identifier PXD035916 and https://doi.org/10.6019/PXD035916. All deposited resources and information will be available once the paper is open to the public.
