# Supplementary material for: Proximity-dependent biotin labeling in testicular germ cells identified TESMIN-associated proteins
Source: Sci Rep. 2022 Dec 23;12:22198. doi: 10.1038/s41598-022-26501-7 (PMC9789103; doi:10.1038/s41598-022-26501-7)
Supplement: Supplementary file 3 — Supplementary Information 1. [file 41598_2022_26501_MOESM3_ESM.pdf]

Table S1. Material used in this study

Mouse lines	BRC	CARD
B6D2-Mtl5<em1Osb>	#RBRC09987	#CARD2454
B6D2-Mtl5<em1Osb> Tg(Clgn-Tesmin/BioID2/3xFLAG)1Osb	#RBRC11222	#CARD3026
B6D2-Tg(Clgn-Tesmin/TurboID/3xFLAG)1Osb	#RBRC11665	#CARD3222

Plasmids	Addgene	RDB
pCAG1.1	#173685	#19246
pClgn1.1	#173686	#19247
pCAG-3xFLAG-BioID2-MCS	#186811	#19657
pCAG-3xFLAG-TurboID-MCS	#186812	#19658
pCAG-MCS-BioID2-3xFLAG	#186813	#19659
pCAG-MCS-TurboID-3xFLAG	#186814	#19660
pCAG-Tesmin-BioID2-3xFLAG	#186815	#19661
pClgn-Tesmin-BioID2-3xFLAG	#186816	#19662
pClgn-Tesmin-TurboID-3xFLAG	#186817	#19963

Primers	Sequence (5'-3')
Tesmin em1 fw	accacgctcggctgagtttc
Tesmin em1 rv	acaagagagagtcgcttgac
Clgn promoter fw	ttgagcgggccgcttgcgcactgg
BioID2 *rv*	atgcgctagcgcttcttctcaggctgaac
TurboID *rv*	atgcgctagccttttcggcagaccgcagac

Antibodies	Source	Identifier
Mouse monoclonal anti-FLAG antibody (M2)	Sigma-Aldrich	Cat# F3165
Mouse monoclonal anti-SYCP3	Santa Cruz Biotechnology	Cat# sc-74569
Rabbit polyclonal anti-gamma-H2A.X	Abcam	Cat# ab11174
Rabbit polyclonal anti-MYBL1	Sigma-Aldrich	Cat# HPA008791
Rabbit polyclonal anti-LIN9	Sigma-Aldrich	Cat# HPA030241
Rabbit polyclonal anti-DVL2	Sigma-Aldrich	Cat# HPA022914
Goat anti-rabbit IgG-HRP	Jackson ImmunoResearch	Cat# 111-036-045
Goat anti-mouse IgG-HRP	Jackson ImmunoResearch	Cat# 115-036-062
Goat anti-mouse IgG-Alexa Fluor 546	Thermo Fisher Scientific	Cat# A11017
Goat anti-rabbit IgG-Alexa Fluor 488	Thermo Fisher Scientific	Cat# A11070
Goat anti-rabbit IgG-Alexa Fluor 546	Thermo Fisher Scientific	Cat# A11071

Table S2. Numerical data underline graphs

Fig. 1D

male mice	pups/plug																average	SD
WT	8	9	1	11	9	10	12	5	9	9								
WT	10	9	9	12	13	12	0	10	10								9.17857143	3.0
WT	8	9	10	10	12	10	8	12	10									
Tesmin$^{em1/em1}$	0	0	0	0	0	0	0	0	0	0	0	0	0	0	0	0		
Tesmin$^{em1/em1}$	0	0	0	0	0	0	0	0	0								0	-
Tesmin$^{em1/em1}$	0	0	0	0	0	0	0	0	0									
Tesmin$^{wt/em1}$, BioID2	8	9	8	13	11	11	10											
Tesmin$^{wt/em1}$, BioID2	7	8	12	11	11	12	5	11									8.59090909	4.0
Tesmin$^{wt/em1}$, BioID2	0	0	0	9	10	13	10											
Tesmin$^{em1/em1}$, BioID2	8	9	8	11	2	8												
Tesmin$^{em1/em1}$, BioID2	8	10	12	7	1	0											8.18181818	3.3
Tesmin$^{em1/em1}$, BioID2	9	9	10	12	8													
Tesmin$^{em1/em1}$, BioID2	9	9	10	12	8													
Tesmin$^{wt/em1}$, TurboID	10	9	8	9	6	11	3	11										
Tesmin$^{wt/em1}$, TurboID	2	8	8	8	3	10	6	12									8.03333333	2.4
Tesmin$^{wt/em1}$, TurboID	9	8	8	10	6	10	6	9										
Tesmin$^{wt/em1}$, TurboID	7	7	7	10	10	10												
Tesmin$^{em1/em1}$, TurboID	0	0	0	0	0	0	0	0	0	0	0	0						
Tesmin$^{em1/em1}$, TurboID	0	0	0	0	0	0	0	0	0	0	0						0	-
Tesmin$^{em1/em1}$, TurboID	0	0	0	0	0	0	0	0	0	0	0	0						

Fig. 1G

male mice	age	body weight (g)	testis weight (g)		Testis/ body weight	average	SD
Tesmin$^{wt/em1}$	12 weeks	37.34	0.12398	0.11788	0.0032386		
Tesmin$^{wt/em1}$	12 weeks	28.41	0.10152	0.10315	0.0036021		
Tesmin$^{wt/em1}$	12 weeks	31.72	0.09994	0.0937	0.0030523	0.0034749	0.0003148
Tesmin$^{wt/em1}$	12 weeks	27.92	0.10353	0.10797	0.0037876		
Tesmin$^{wt/em1}$	12 weeks	30.52	0.1145	0.11098	0.003694		
Tesmin$^{em1/em1}$	12 weeks	28.56	0.03022	0.00307	0.0005828		
Tesmin$^{em1/em1}$	12 weeks	32.32	0.02594	0.02578	0.0008001		
Tesmin$^{em1/em1}$	12 weeks	29.52	0.03205	0.02927	0.0010386		
Tesmin$^{em1/em1}$	12 weeks	29.03	0.02761	0.02929	0.00098	0.0009944	0.0002834
Tesmin$^{em1/em1}$	12 weeks	35.2	0.03437	0.03284	0.0009547		
Tesmin$^{em1/em1}$	13 weeks	27.48	0.02847	0.03204	0.001101		
Tesmin$^{em1/em1}$	13 weeks	23.14	0.0342	0.0354	0.0015039		
Tesmin$^{wt/em1}$, BioID2	12 weeks	23.72	0.10301	0.09452	0.0041638		
Tesmin$^{wt/em1}$, BioID2	13 weeks	30.22	0.10928	0.10776	0.003591	0.0038884	0.00042
Tesmin$^{wt/em1}$, BioID2	12 weeks	30.2	0.10898	0.10076	0.0034725		
Tesmin$^{wt/em1}$, BioID2	13 weeks	26.01	0.11617	0.10889	0.0043264		
Tesmin$^{em1/em1}$, BioID2	12 weeks	29.44	0.07302	0.07598	0.0025306		
Tesmin$^{em1/em1}$, BioID2	12 weeks	25.73	0.07457	0.07632	0.0029322		
Tesmin$^{em1/em1}$, BioID2	10 weeks	28.08	0.10071	0.10641	0.003688	0.0027518	0.0006761
Tesmin$^{em1/em1}$, BioID2	10 weeks	28.34	0.05498	0.05601	0.0019582		
Tesmin$^{em1/em1}$, BioID2	13 weeks	34.13	0.07275	0.07145	0.0021125		
Tesmin$^{em1/em1}$, BioID2	13 weeks	22.5	0.07581	0.07221	0.0032893		
Tesmin$^{wt/em1}$, TurboID	12 weeks	29.12	0.10802	0.1138	0.0038087		
Tesmin$^{wt/em1}$, TurboID	12 weeks	31.22	0.1187	0.11874	0.0038027	0.0039124	0.0001379
Tesmin$^{wt/em1}$, TurboID	12 weeks	28.04	0.11937	0.11028	0.004095		
Tesmin$^{wt/em1}$, TurboID	12 weeks	33.35	0.12918	0.13382	0.003943		
Tesmin$^{em1/em1}$, TurboID	19 weeks	24.73	0.03442	0.03095	0.0013217		
Tesmin$^{em1/em1}$, TurboID	19 weeks	30.05	0.0338	0.03565	0.0011556		
Tesmin$^{em1/em1}$, TurboID	21 weeks	33.79	0.05778	0.05509	0.0016702		
Tesmin$^{em1/em1}$, TurboID	12 weeks	33.6	0.04879	0.0467	0.001421	0.0014744	0.0002126
Tesmin$^{em1/em1}$, TurboID	12 weeks	29.77	0.04891	0.05181	0.0016916		
Tesmin$^{em1/em1}$, TurboID	12 weeks	29.39	0.04585	0.0474	0.0015864		

Fig. S2B

male mice	tubule area (cm^2)	TUNEL + cells			
		total	stage II-V	stageVI-X	stage XI-I
Tesmin$^{wt/em1}$	7.6	109	19	1	89
Tesmin$^{wt/em1}$	7.6	43	9	1	33
Tesmin$^{wt/em1}$	6.3	41	3	0	38
Tesmin$^{em1/em1}$	3.6	239	197	0	42
Tesmin$^{em1/em1}$	3.5	292	283	0	9
Tesmin$^{em1/em1}$	4.1	323	298	0	25
Tesmin$^{wt/em1}$, BioID2	8.4	81	12	1	68
Tesmin$^{wt/em1}$, BioID2	6.9	34	2	3	29
Tesmin$^{wt/em1}$, BioID2	9.5	49	1	13	35
Tesmin$^{em1/em1}$, BioID2	4.9	175	39	100	36
Tesmin$^{em1/em1}$, BioID2	5.4	235	74	45	116
Tesmin$^{em1/em1}$, BioID2	4.4	144	44	49	51
Tesmin$^{wt/em1}$, TurboID	8.7	40	1	1	38
Tesmin$^{wt/em1}$, TurboID	11.5	30	0	5	25
Tesmin$^{wt/em1}$, TurboID	7.1	66	5	2	59
Tesmin$^{em1/em1}$, TurboID	4	135	74	18	43
Tesmin$^{em1/em1}$, TurboID	5.4	223	128	16	79
Tesmin$^{em1/em1}$, *TurboID*	6	267	155	39	73

Fig. S2D

male mice	# of cells		
	Initiated	Intermediate	Completed
Tesmin$^{wt/em1}$	43	41	120
Tesmin$^{wt/em1}$	39	29	158
Tesmin$^{wt/em1}$	32	24	149
Tesmin$^{em1/em1}$	43	88	55
Tesmin$^{em1/em1}$	19	22	58
Tesmin$^{em1/em1}$	28	66	103
Tesmin$^{wt/em1}$, BioID2	28	16	75
Tesmin$^{wt/em1}$, BioID2	19	23	91
Tesmin$^{wt/em1}$, BioID2	23	22	93
Tesmin$^{em1/em1}$, BioID2	62	50	115
Tesmin$^{em1/em1}$, BioID2	29	40	160
Tesmin$^{em1/em1}$, BioID2	32	46	98
Tesmin$^{wt/em1}$, TurboID	23	18	63
Tesmin$^{wt/em1}$, TurboID	13	20	80
Tesmin$^{wt/em1}$, TurboID	33	26	102
Tesmin$^{em1/em1}$, TurboID	45	89	70
Tesmin$^{em1/em1}$, TurboID	21	63	59
Tesmin$^{em1/em1}$, *TurboID*	31	73	95

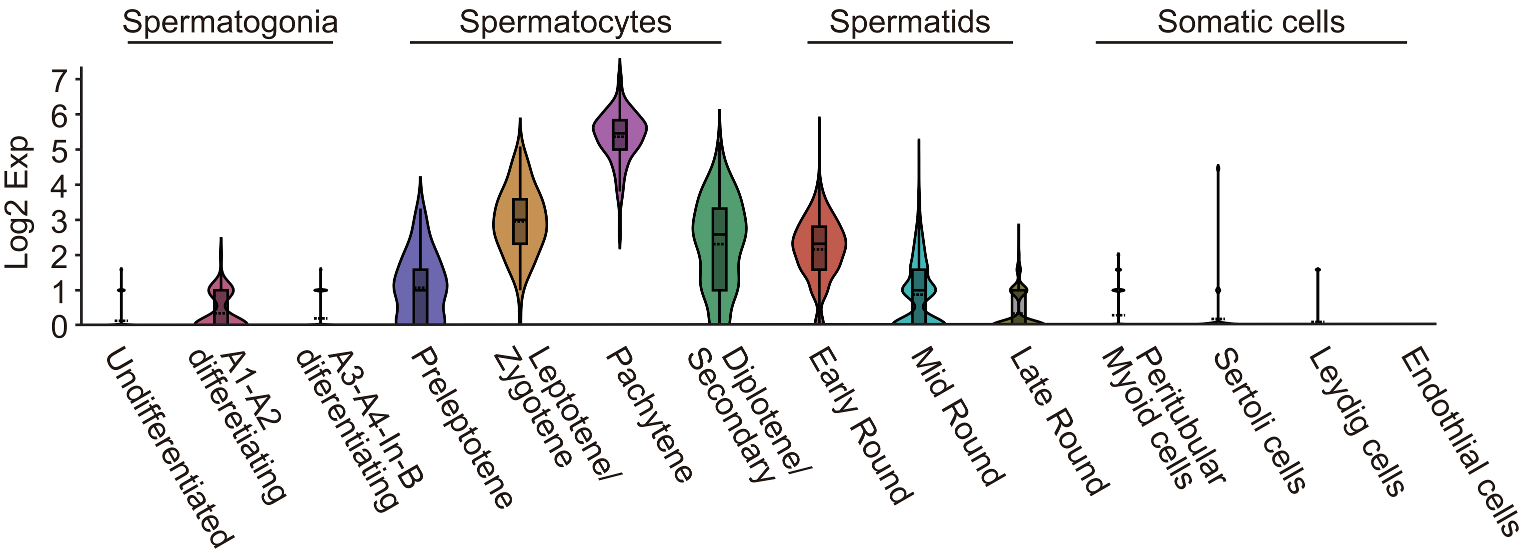

Figure S1. The *Tesmin* expression profile.
The *Tesmin* expression profile between testicular cells based on published single-cell RNA sequencing data, visualized by 10 x genomics Loupe Browser.

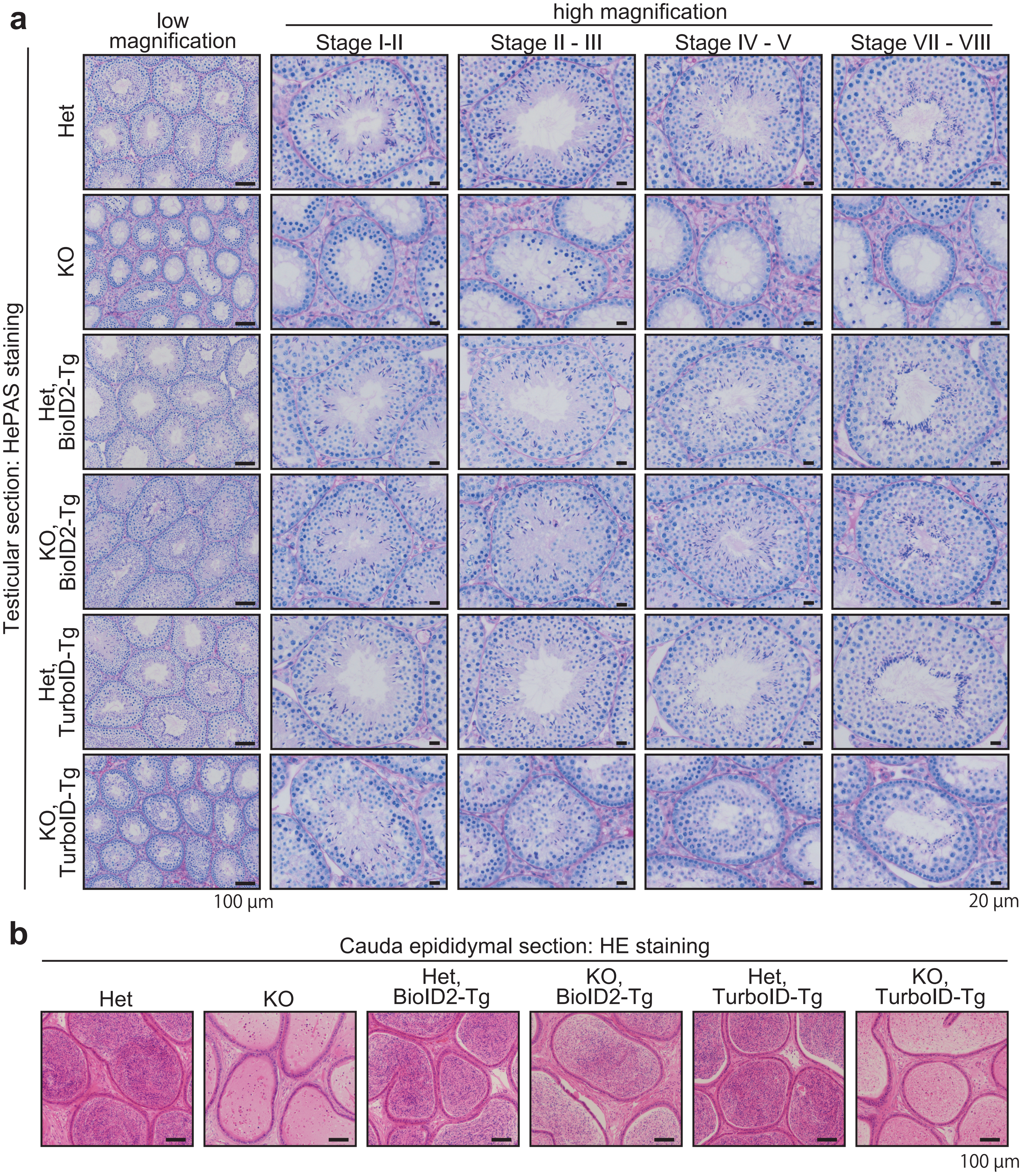

Figure S2. Histological analysis of transgenic mice.
(a) PAS staining of seminiferous tubules of adult mice (n=3). The seminiferous epithelium cycle was determined by germ cell position and nuclear morphology. (b) HE staining of cauda epididymal sections from adult mice (n=3).

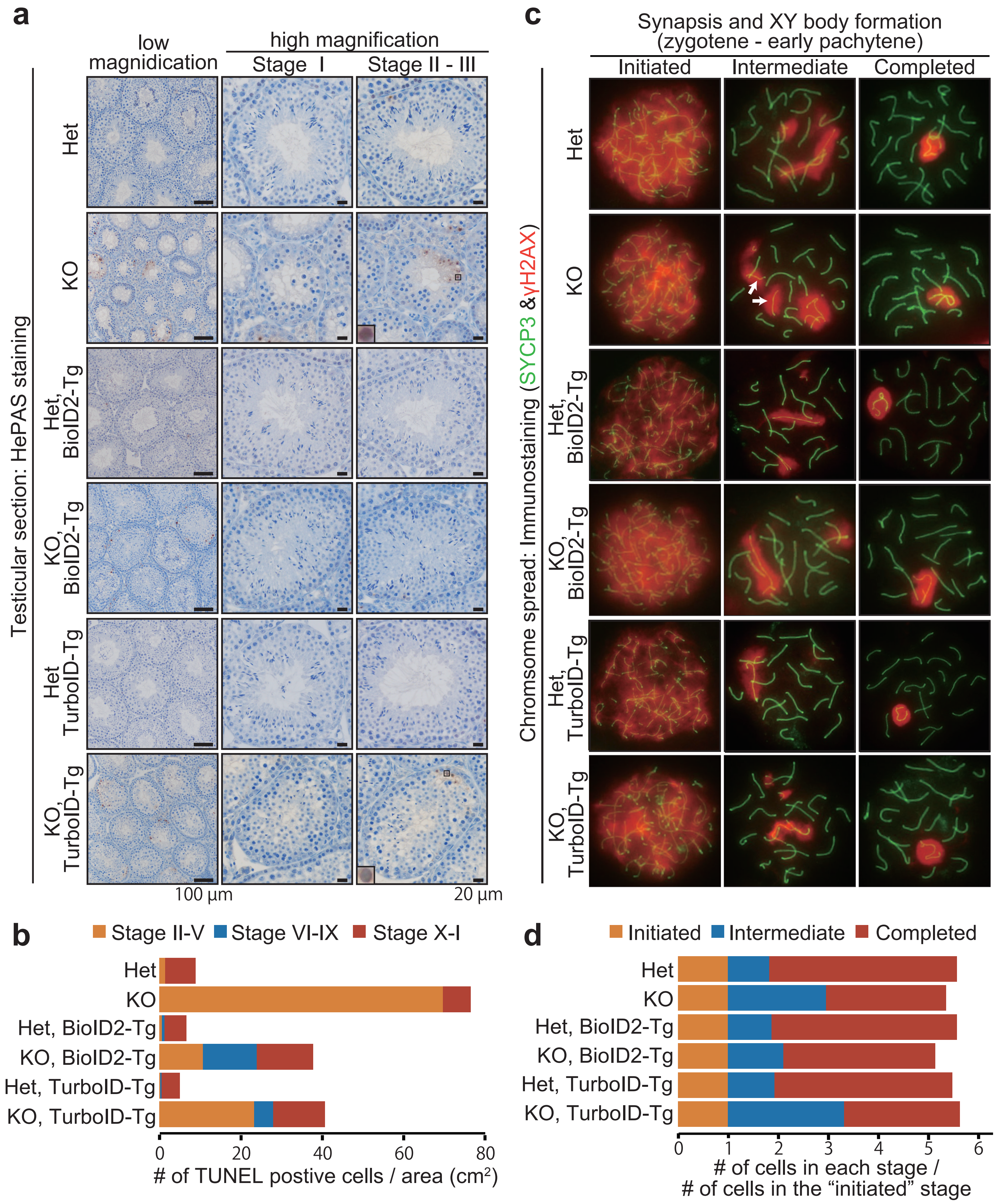

Figure S3. Histological and cytological analysis of transgenic mice.
(a) TUNEL staining of seminiferous tubules of adult mice counterstained with hematoxylin. Three males were examined (n=3). (b) The number of TUNEL positive cells stained in a. The seminiferous epithelial stages were roughly determined by the arrangement and nuclear morphology of the first layer of germ cells (spermatogonia and leptotene/zygotene spermatocytes). (c) Immunostaining of spread nuclei from prophase spermatocytes collected from adult mice (n=3). Zygotene and early pachytene spermatocytes were categorized into three groups: initiated (0-50% synapsis), Intermediated (50-100% synapsis), completed (100% synapsis and XY body formation). (d) The number of cells in each category in c is normalized by the number of cells in the "initiated" group.

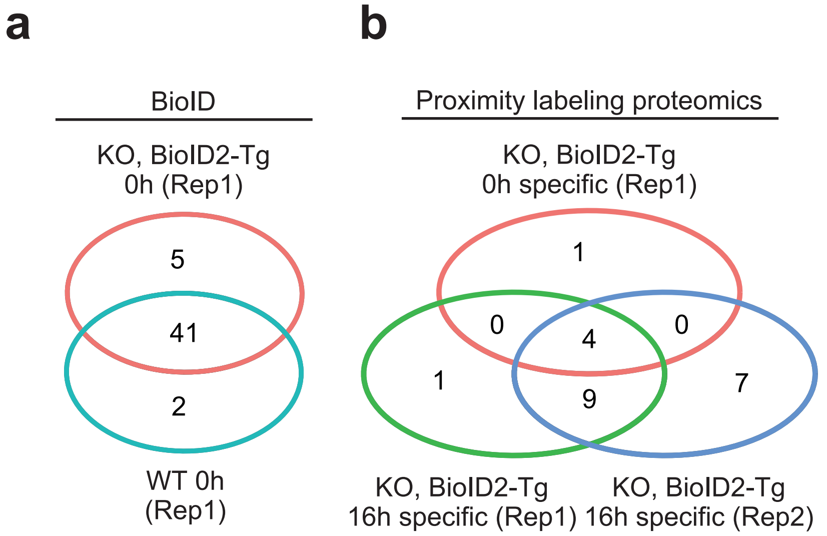

c

Accssion Number	Protein	Quatiative Value (Nomalized Total Spectra) BioID2 0h Rep1	BioID2 16h Rep1	BioID2 16h Rep2
Q8C735	Lin9	19.8	29.8	15.5
P51960	MYBL1	8.4	28.6	21.4
Q9D9C7*	-	8.4	11.9	7.4
Q60838	DVL2	7.3	10.7	3.7
Q9WTJ6	TESMIN	0	13.1	0.7
P45481	CREBBP	0	3.6	2.9
Q9D8N6	LIN37	0	3.6	2.2
P80316	CCT5	0	3.6	0.7
P51141	DVL1	0	2.4	5.2
Q562E2	KCTD19	0	1.2	2.9
Q9EQ00	RONPN1L	0	1.2	2.2
Q8BU14	SEC62	0	1.2	2.2
Q8K310	MATR3	0	1.2	1.5

Q9D9C7*: Uncharacterized protein C3orf62 homolog

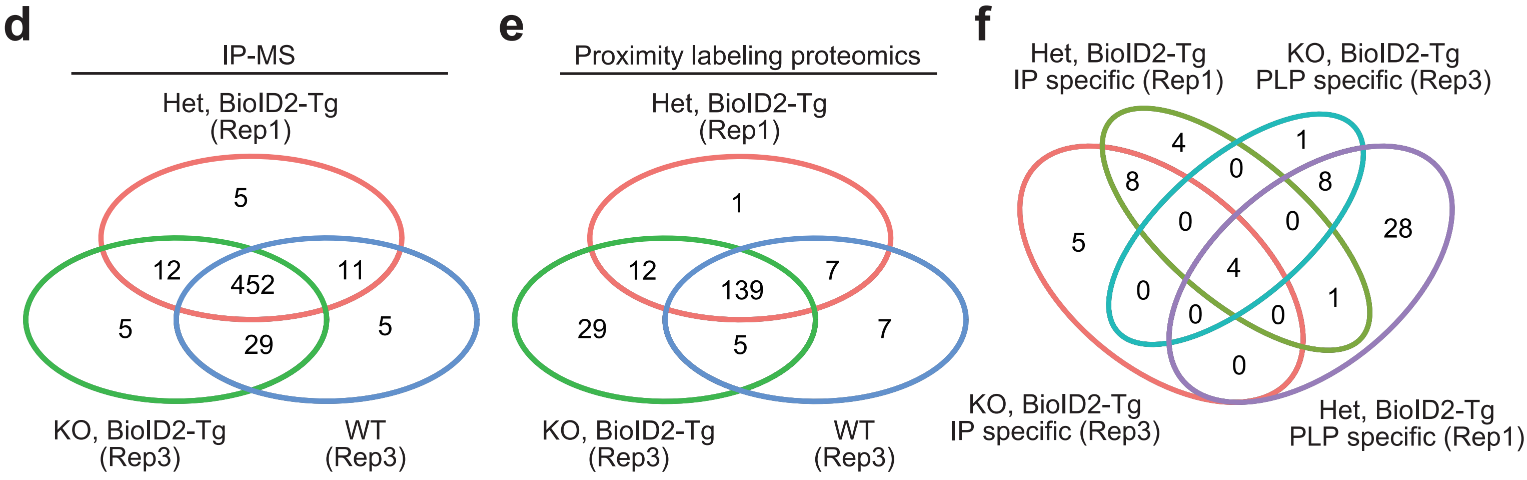

g

Accssion Number	Protein	Quatiative Value (Nomalized Total Spectra) KO, Tg IP	Het, Tg IP	KO, Tg PLP	Het, Tg PLP
Q8C735	LIN9	30.7	20.9	20.8	18.8
Q9WTJ6	TESMIN	27.8	52.3	2.0	5.2
B2RWS6	EP300	24.8	18.8	0	0
P51960	MYBL1	23.8	12.6	10.9	4.2
P45481	CREBBP	16.9	7.3	0	0
Q9D9C7*	-	11.9	6.3	0	0
Q9R1Z8	SORBS3	4.0	6.3	0	0
Q8CD94	LIN52	4.0	4.2	0	0
Q9D8N6	LIN37	4.0	3.1	0	0
Q79IV5	SYNM	4.0	1.0	0	0

Q9D9C7*: Uncharacterized protein C3orf62 homolog

Accssion Number	Protein	Quatiative Value (Nomalized Total Spectra) KO, Tg IP	Het, Tg IP	KO, Tg PLP	Het, Tg PLP
P05480	SRC	1.0	2.1	0	0
Q3UYG1	CCDC160	1.0	2.1	0	0
Q9CYG7	TOMM34	0	4.2	2.0	0
P54869	HMGCS2	0	0	8.9	5.2
Q8BFR5	CAPZB	0	0	6.9	1.0
P51141	DVL1	0	0	6.0	10.5
Q8BFr5	TUFM	0	0	4.0	2.1
Q60838	DVL2	0	0	2.0	10.5
Q14AX6	CDK12	0	0	2.0	1.0
Q88665	BRD7	0	0	1.0	2.1
Q8R361	RAB11FIP5	0	0	1.0	2.1

Figure S4. Additional proteomic analysis using BioID2-Tg mice.
(a) Venn diagram of BioID result using KO, BioI2-Tg mice without incubation in biotin supplemented medium. (b) Venn diagram of specific spectrum in d and Fig. 2b to compare 16 h and 0 h incubation in biotin supplemented medium. (c) Summary of specific spectra. Proteins detected at least twice in b were extracted. The whole data is available in Dataset S1. (d and e) Venn diagram of IP-Mass (d) and BioID (e) analysis using BioID2-Tg mice with heterozygous and homozygous KO allele. (f) Venn diagram of the specific spectrum (not detected in WT) in d and e. PLP: Proximity labeling proteomics. (g) Summary of specific spectra. Proteins detected at least twice in f were extracted. PLP: Proximity labeling proteomics. The whole data is available in Dataset S1.

a

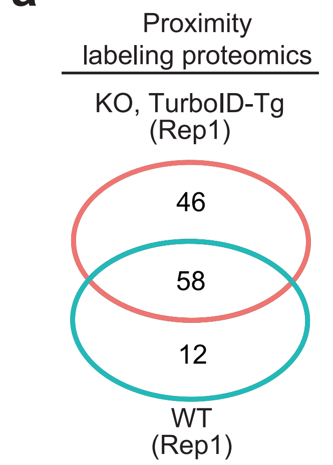

b

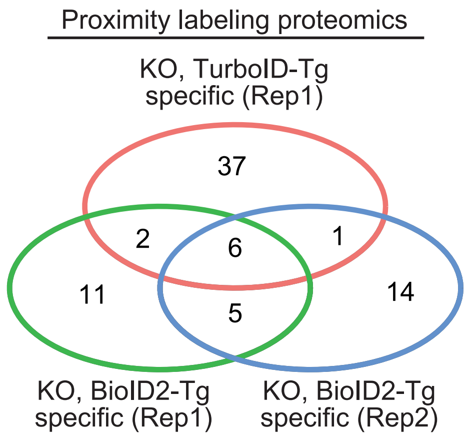

c

Accssion Number	Protein	Quatiative Value (Nomalized Total Spectra)		
		TurboID	BioID2	
			Rep1	Rep2
Q9D9C7		16.0	11.9	7.4
Q8C735	LIN9	10.6	29.8	15.5
P51960	MYBL1	8.9	28.6	21.4
A2A9R3	MAGEB4	3.5	0	1.5
Q9WTJ6	TESMIN	1.8	13.1	0.7
Q562E2	KCTD19	1.8	1.2	2.9
Q60838	DVL2	0	10.7	3.7
P45481	CREBBP	0	3.6	2.9
Q9D8N6	LIN37	0	3.6	2.2
P80316	CCT5	0	3.6	0.7
P51141	DVL1	0	2.4	5.2
Q9EQ00	ROPN1L	0	1.2	2.2
Q8BU14	SEC62	0	1.2	2.2
Q8K310	MATR3	0	1.2	1.5

d

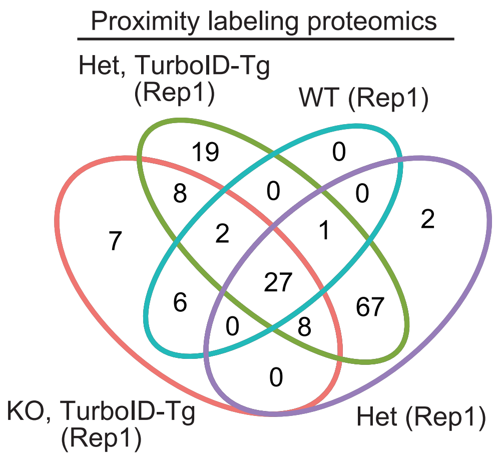

e

Accssion Number	Protein	Quatiative Value (Nomalized Total Spectra)	
		KO, Tg	Het, Tg
Q9D9C7		16.0	6.4
Q01320	TOP2A	11.5	0
Q8C735	LIN9	10.6	30.6
P51960	MYBL1	8.9	24.2
P42932	CCT8	4.4	33.8
Q689Z5	SBNO1	3.5	15.3
A2A9R3	MAGEB4	3.5	0
B2RWS6	EP300	3.5	0
Q99MV1	TDRD1	3.5	0
Q562E2	KCTD19	1.8	8.1
Q9WTJ6	TESMIN	1.8	6.4
Q9DCD2	XAB2	1.8	5.6
Q00547	HMMR	1.8	0
Q8BT07	CEP55	1.8	0
Q8CD94	LIN52	1.8	0
E9Q4Z2	ACACB	0	33.0
Q9D620	RAB11FIP1	0	7.3

Accssion Number	Protein	Quatiative Value (Nomalized Total Spectra)	
		KO, Tg	Het, Tg
Q9QZ29	IGBP1B	0	7.3
Q8BG79	CWF19L2	0	6.4
Q8K2K6	AGFG1	0	5.6
Q0GGX2	ZFP541	0	4.8
P11983	TCP1	0	4.0
DC2I2_MOUSE	-	0	3.2
O54826	MLLT10	0	2.4
Q0VBL3	RBM15	0	2.4
Q5S003	SPAG17	0	2.4
Q99MR8	MCCC1	0	2.4
Q9Z315	SART1	0	2.4
O70311	NMT2	0	1.6
P97386	LIG3	0	1.6
Q8BU14	SEC62	0	1.6
Q8C176	TAF2	0	1.6
Q8CDE2	CCIN	0	1.6
Q9QVN7	TCEA2	0	1.6

Figure S5. Proteomic analysis using TurboID-Tg mice.
(a) Venn diagram of proximity labeling proteomics result using WT, TurboID-Tg mice. (b) Venn diagram of specific spectrum in a and Fig. 2b to compare BioID2 and TurboID. (c) Summary of specific spectra. Proteins detected at least twice (minimum total spectrum: 2) in b were extracted. The whole data is available in Dataset S1. (d) Venn diagram of proximity labeling proteomics result using WT, TurboID-Tg mice with heterozygous and homozygous KO alleles. (e) Summary of specific spectra in d. Proteins detected only in TurboID-Tg mice with heterozygous or homozygous KO allele were extracted. The whole data is available in Dataset S1.

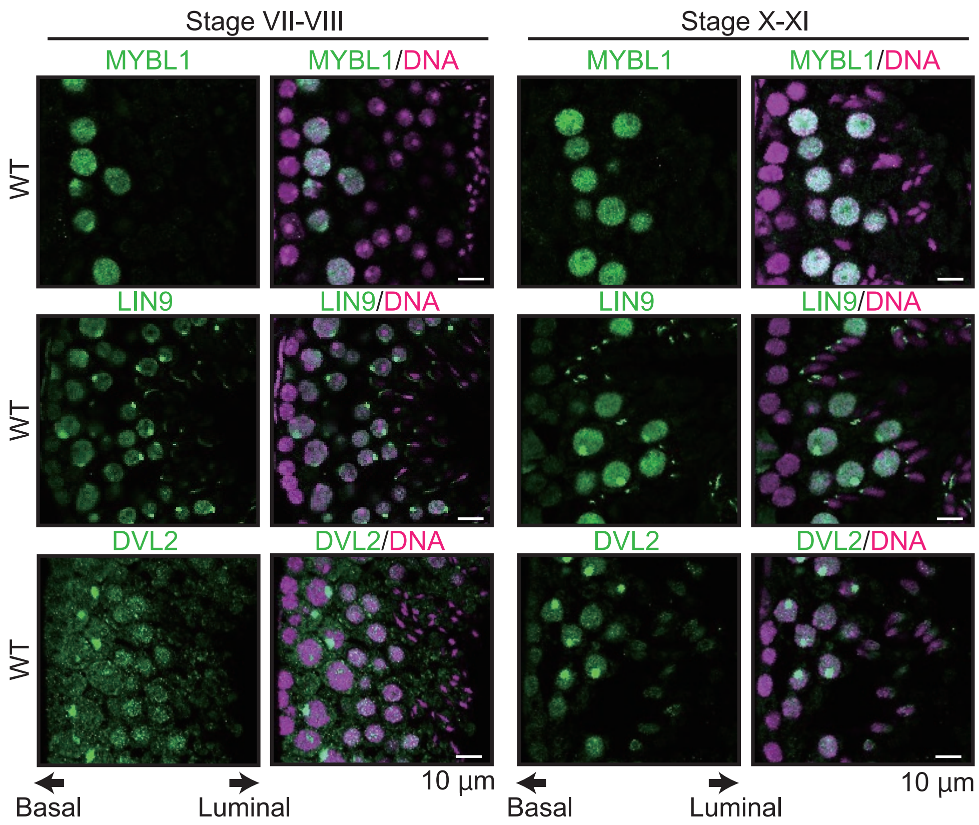

Figure S6. Immunostaining of testicular sections.
Immunostaining of testis sections using anti-MYBL1, -LIN9, and -DVL2 antibodies. The seminiferous epithelium cycle was determined by cell position, nuclear morphology, and morphology of the acrosome stained with AlexaFlour 568-conjugated lectin PNA.

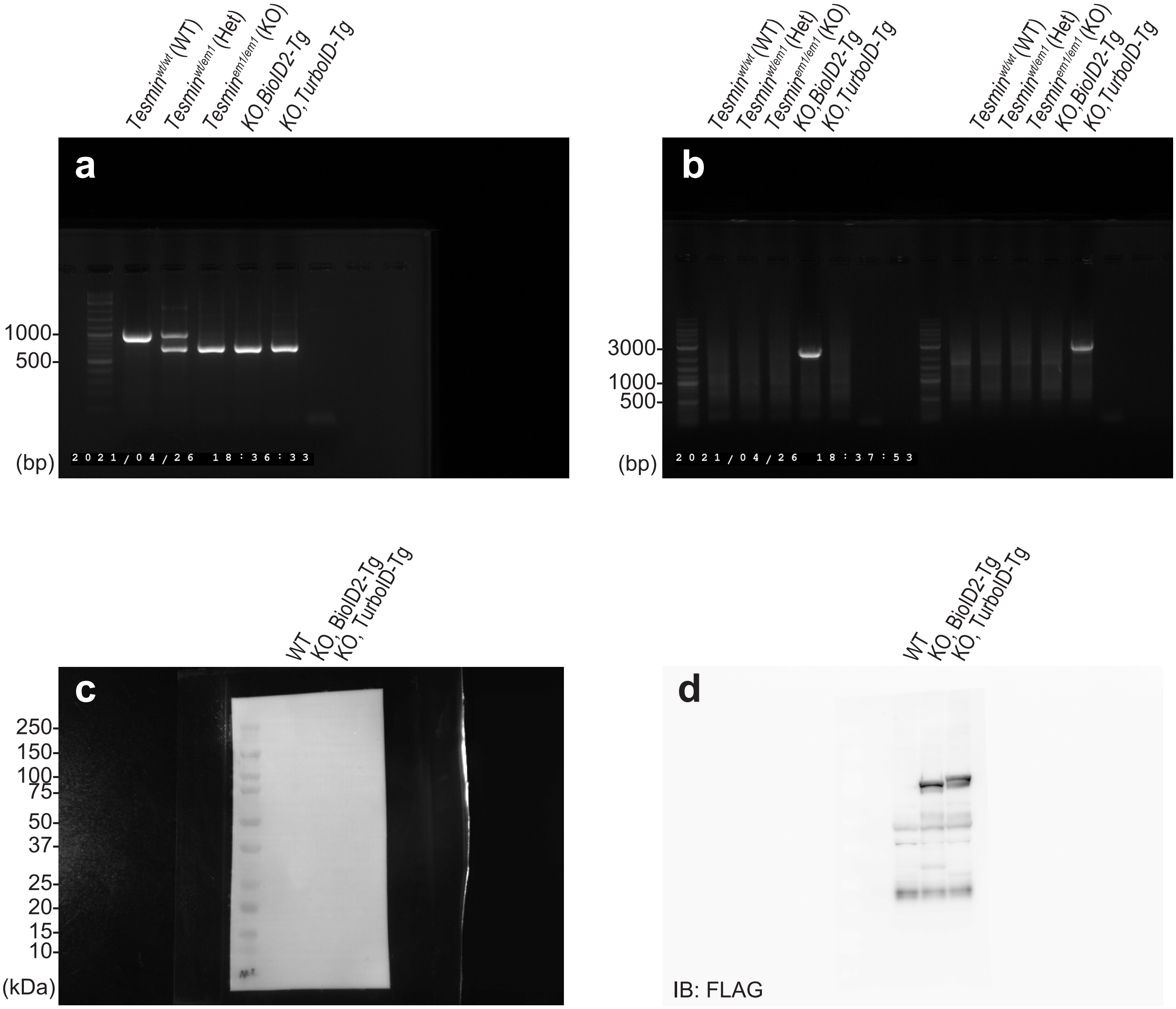

Figure S7. Unprocessed and original images of gels and blots provided in Figure 1.
(a and b) The original images of Fig. 1b. An example of genotyping for *Tesmin*^{em1} (a) and BioID2 and TurboID-Tg (b). (c and d) The original images of Fig. 1c. The bright field image for size marker (c) and chemiluminescent image for FLAG immunoblotting (d).

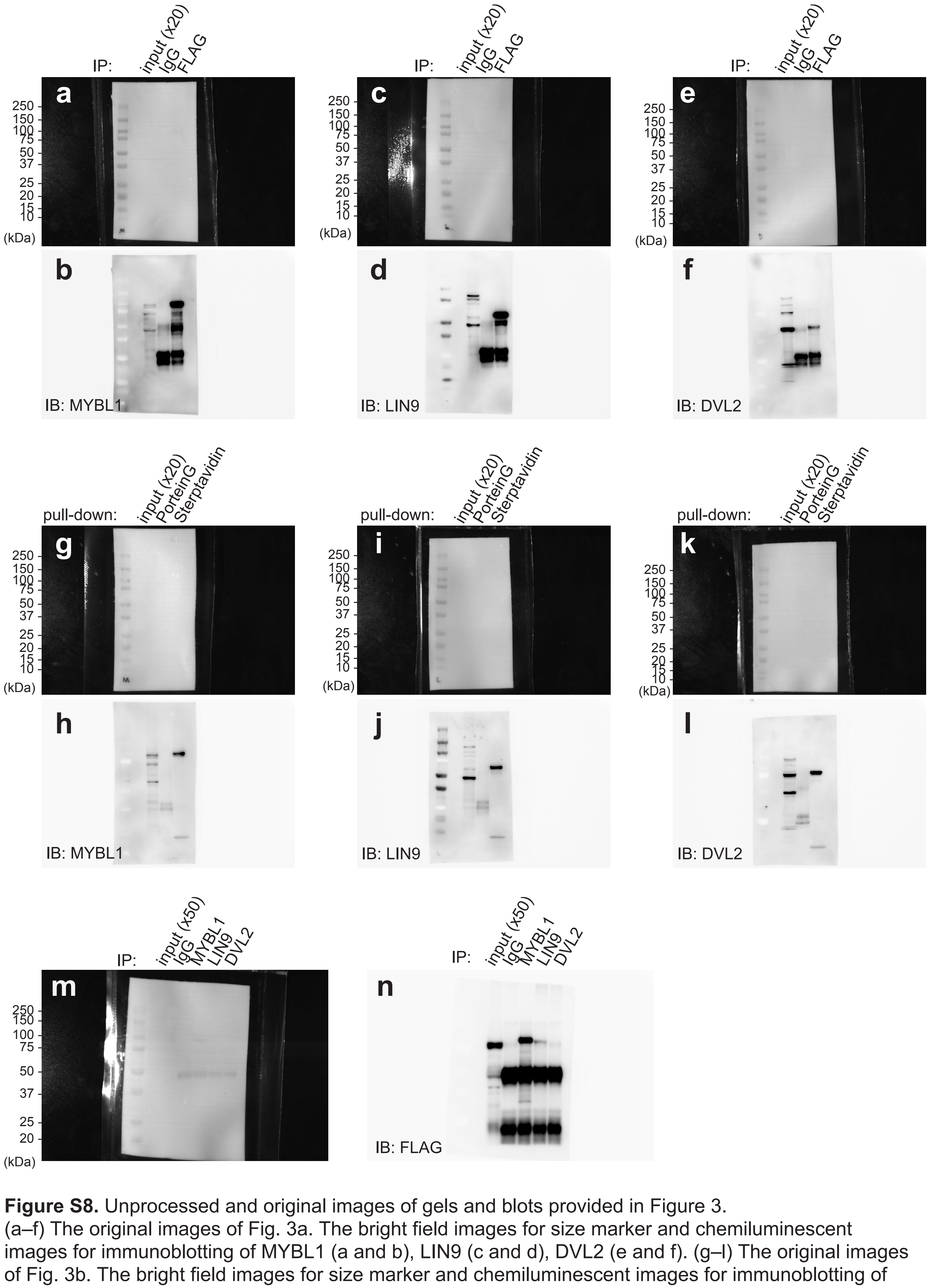

Figure S8. Unprocessed and original images of gels and blots provided in Figure 3. (a–f) The original images of Fig. 3a. The bright field images for size marker and chemiluminescent images for immunoblotting of MYBL1 (a and b), LIN9 (c and d), DVL2 (e and f). (g–l) The original images of Fig. 3b. The bright field images for size marker and chemiluminescent images for immunoblotting of MYBL1 (g and h), LIN9 (i and j), DVL2 (k and l). (m and n) The original images of Fig. 3c. The bright field images for size marker (m) and chemiluminescent images for immunoblotting of FLAG (n).
